# Supplementary material for: Modeling glioblastoma heterogeneity as a dynamic network of cell states
Source: Mol Syst Biol. 2021 Sep 16;17(9):e10105. doi: 10.15252/msb.202010105 (PMC8444284; doi:10.15252/msb.202010105)
Supplement: Supplementary file 6 — Source Data for Figure 5 [file MSB-17-e10105-s004.zip › Figure5A_sourcedata/GSEA_3071/nosigdb_stateV.GseaPreranked.1622795159954/DOROSZKO_U3054MG_CLUSTER_2.html]

Details for gene set DOROSZKO\_U3054MG\_CLUSTER\_2[GSEA]

|  || Dataset | state43071 |
| Phenotype | NoPhenotypeAvailable |
| Upregulated in class | na\_neg |
| GeneSet | DOROSZKO\_U3054MG\_CLUSTER\_2 |
| Enrichment Score (ES) | -0.56692225 |
| Normalized Enrichment Score (NES) | -3.748354 |
| Nominal p-value | 0.0 |
| FDR q-value | 0.0 |
| FWER p-Value | 0.0 |
Table: GSEA Results Summary

  

Fig 1: Enrichment plot: DOROSZKO\_U3054MG\_CLUSTER\_2      
 Profile of the Running ES Score & Positions of GeneSet Members on the Rank Ordered List

  

| PROBE | GENE SYMBOL | GENE\_TITLE | RANK IN GENE LIST | RANK METRIC SCORE | RUNNING ES | CORE ENRICHMENT || 1 | IGFBP3 |  |  | 78 | 1.437 | -0.0095 | No |
| 2 | SCRG1 |  |  | 87 | 1.404 | -0.0053 | No |
| 3 | CXCL14 |  |  | 100 | 1.362 | -0.0020 | No |
| 4 | RASSF2 |  |  | 107 | 1.351 | 0.0024 | No |
| 5 | PDGFRA |  |  | 109 | 1.343 | 0.0078 | No |
| 6 | NRP2 |  |  | 184 | 1.204 | -0.0019 | No |
| 7 | NGFR |  |  | 197 | 1.177 | 0.0006 | No |
| 8 | SULF2 |  |  | 236 | 1.131 | -0.0023 | No |
| 9 | DCX |  |  | 258 | 1.107 | -0.0019 | No |
| 10 | PLPP3 |  |  | 323 | 1.042 | -0.0103 | No |
| 11 | RGS16 |  |  | 348 | 1.018 | -0.0108 | No |
| 12 | JAM2 |  |  | 351 | 1.015 | -0.0070 | No |
| 13 | ZFHX4 |  |  | 362 | 0.996 | -0.0048 | No |
| 14 | CDKN1A |  |  | 363 | 0.993 | -0.0007 | No |
| 15 | FRMD4B |  |  | 381 | 0.968 | -0.0001 | No |
| 16 | SPRY1 |  |  | 389 | 0.959 | 0.0025 | No |
| 17 | CLU |  |  | 464 | 0.900 | -0.0084 | No |
| 18 | CDK6 |  |  | 605 | 0.798 | -0.0330 | No |
| 19 | PLPPR5 |  |  | 638 | 0.780 | -0.0361 | No |
| 20 | C16orf45 |  |  | 655 | 0.771 | -0.0361 | No |
| 21 | ADAM22 |  |  | 716 | 0.740 | -0.0449 | No |
| 22 | ADAMTS9 |  |  | 726 | 0.733 | -0.0436 | No |
| 23 | SSBP4 |  |  | 735 | 0.729 | -0.0422 | No |
| 24 | STIM2 |  |  | 742 | 0.727 | -0.0404 | No |
| 25 | SHROOM3 |  |  | 747 | 0.724 | -0.0382 | No |
| 26 | TCF4 |  |  | 757 | 0.720 | -0.0370 | No |
| 27 | TMEM59L |  |  | 768 | 0.715 | -0.0360 | No |
| 28 | BMP7 |  |  | 777 | 0.711 | -0.0346 | No |
| 29 | TGIF1 |  |  | 840 | 0.683 | -0.0441 | No |
| 30 | KLHL13 |  |  | 853 | 0.677 | -0.0437 | No |
| 31 | FGFR1 |  |  | 862 | 0.673 | -0.0425 | No |
| 32 | C11orf96 |  |  | 876 | 0.668 | -0.0423 | No |
| 33 | SOX9 |  |  | 905 | 0.658 | -0.0451 | No |
| 34 | VEGFA |  |  | 926 | 0.649 | -0.0464 | No |
| 35 | NAB1 |  |  | 929 | 0.648 | -0.0441 | No |
| 36 | GAD1 |  |  | 947 | 0.641 | -0.0448 | No |
| 37 | FARP1 |  |  | 956 | 0.638 | -0.0438 | No |
| 38 | AC009041.2 |  |  | 995 | 0.621 | -0.0487 | No |
| 39 | COL1A2 |  |  | 1028 | 0.614 | -0.0526 | No |
| 40 | TLE2 |  |  | 1074 | 0.601 | -0.0590 | No |
| 41 | MRAS |  |  | 1108 | 0.590 | -0.0631 | No |
| 42 | TRIL |  |  | 1127 | 0.586 | -0.0643 | No |
| 43 | SHOX2 |  |  | 1128 | 0.586 | -0.0618 | No |
| 44 | PDS5B |  |  | 1140 | 0.580 | -0.0616 | No |
| 45 | KCNE4 |  |  | 1272 | 0.539 | -0.0854 | No |
| 46 | ITPR1 |  |  | 1282 | 0.537 | -0.0850 | No |
| 47 | SPRY4 |  |  | 1283 | 0.536 | -0.0827 | No |
| 48 | EFEMP2 |  |  | 1300 | 0.532 | -0.0837 | No |
| 49 | NES |  |  | 1325 | 0.527 | -0.0863 | No |
| 50 | CTTNBP2 |  |  | 1374 | 0.513 | -0.0937 | No |
| 51 | LTBP4 |  |  | 1436 | 0.496 | -0.1038 | No |
| 52 | OLFM2 |  |  | 1482 | 0.486 | -0.1107 | No |
| 53 | PLXDC1 |  |  | 1487 | 0.483 | -0.1095 | No |
| 54 | RAMP2 |  |  | 1559 | 0.468 | -0.1216 | No |
| 55 | PLEKHH2 |  |  | 1595 | 0.461 | -0.1267 | No |
| 56 | CTDSPL |  |  | 1598 | 0.461 | -0.1252 | No |
| 57 | ZEB1 |  |  | 1605 | 0.459 | -0.1244 | No |
| 58 | ZNF331 |  |  | 1630 | 0.453 | -0.1273 | No |
| 59 | DAAM1 |  |  | 1653 | 0.445 | -0.1299 | No |
| 60 | C1R |  |  | 1696 | 0.437 | -0.1364 | No |
| 61 | TLE1 |  |  | 1947 | 0.397 | -0.1844 | No |
| 62 | MEST |  |  | 1980 | 0.391 | -0.1891 | No |
| 63 | ETV4 |  |  | 1998 | 0.389 | -0.1909 | No |
| 64 | TCEAL5 |  |  | 2043 | 0.381 | -0.1981 | No |
| 65 | IL17RD |  |  | 2080 | 0.376 | -0.2037 | No |
| 66 | CAPN5 |  |  | 2086 | 0.375 | -0.2031 | No |
| 67 | GPR162 |  |  | 2152 | 0.367 | -0.2145 | No |
| 68 | FAM89B |  |  | 2153 | 0.367 | -0.2130 | No |
| 69 | ST8SIA4 |  |  | 2207 | 0.361 | -0.2220 | No |
| 70 | ANGPTL1 |  |  | 2246 | 0.357 | -0.2281 | No |
| 71 | TMEM158 |  |  | 2340 | 0.343 | -0.2451 | No |
| 72 | MRC2 |  |  | 2378 | 0.338 | -0.2511 | No |
| 73 | ABI3BP |  |  | 2402 | 0.334 | -0.2542 | No |
| 74 | SCARA3 |  |  | 2409 | 0.334 | -0.2541 | No |
| 75 | GLT8D1 |  |  | 2548 | 0.317 | -0.2802 | No |
| 76 | NBEA |  |  | 2562 | 0.315 | -0.2814 | No |
| 77 | ECM1 |  |  | 2564 | 0.315 | -0.2803 | No |
| 78 | CXCL12 |  |  | 2633 | 0.306 | -0.2926 | No |
| 79 | FAM198B |  |  | 2661 | 0.303 | -0.2967 | No |
| 80 | PRRT2 |  |  | 2685 | 0.301 | -0.3000 | No |
| 81 | CLDN1 |  |  | 2767 | 0.293 | -0.3149 | No |
| 82 | CCND2 |  |  | 2876 | 0.282 | -0.3352 | No |
| 83 | PCDH19 |  |  | 2897 | 0.279 | -0.3380 | No |
| 84 | ANKH |  |  | 2938 | 0.275 | -0.3448 | No |
| 85 | CLEC11A |  |  | 2957 | 0.273 | -0.3472 | No |
| 86 | SEZ6L2 |  |  | 2958 | 0.273 | -0.3461 | No |
| 87 | RUNX1T1 |  |  | 2963 | 0.273 | -0.3458 | No |
| 88 | SLC5A3 |  |  | 3032 | 0.266 | -0.3582 | No |
| 89 | ANTXR1 |  |  | 3037 | 0.265 | -0.3579 | No |
| 90 | STX8 |  |  | 3214 | -0.253 | -0.3918 | No |
| 91 | S1PR2 |  |  | 3231 | -0.257 | -0.3939 | No |
| 92 | COL18A1 |  |  | 3251 | -0.259 | -0.3966 | No |
| 93 | ASPH |  |  | 3273 | -0.262 | -0.3997 | No |
| 94 | TNFAIP2 |  |  | 3314 | -0.269 | -0.4065 | No |
| 95 | ECE1 |  |  | 3354 | -0.275 | -0.4131 | No |
| 96 | BNIP3L |  |  | 3381 | -0.278 | -0.4171 | No |
| 97 | LAMA2 |  |  | 3449 | -0.289 | -0.4292 | No |
| 98 | SAV1 |  |  | 3464 | -0.293 | -0.4308 | No |
| 99 | LRRN4CL |  |  | 3531 | -0.305 | -0.4426 | No |
| 100 | PODNL1 |  |  | 3552 | -0.308 | -0.4453 | No |
| 101 | CDC42EP5 |  |  | 3587 | -0.313 | -0.4508 | No |
| 102 | DOCK9 |  |  | 3658 | -0.324 | -0.4634 | No |
| 103 | NUMBL |  |  | 3666 | -0.325 | -0.4634 | No |
| 104 | CDC42EP2 |  |  | 3688 | -0.329 | -0.4662 | No |
| 105 | AFAP1 |  |  | 3717 | -0.334 | -0.4704 | No |
| 106 | TNS1 |  |  | 3761 | -0.342 | -0.4775 | No |
| 107 | TBC1D2B |  |  | 3766 | -0.343 | -0.4769 | No |
| 108 | GLI2 |  |  | 3770 | -0.344 | -0.4760 | No |
| 109 | CYGB |  |  | 3806 | -0.350 | -0.4815 | No |
| 110 | SDCBP |  |  | 3840 | -0.357 | -0.4866 | No |
| 111 | YBX3 |  |  | 3863 | -0.361 | -0.4895 | No |
| 112 | EPB41L2 |  |  | 3911 | -0.369 | -0.4973 | No |
| 113 | ISLR |  |  | 3936 | -0.375 | -0.5005 | No |
| 114 | HIST1H2AC |  |  | 3949 | -0.377 | -0.5013 | No |
| 115 | COL5A3 |  |  | 3975 | -0.383 | -0.5047 | No |
| 116 | COL21A1 |  |  | 3984 | -0.385 | -0.5047 | No |
| 117 | TLN2 |  |  | 4025 | -0.394 | -0.5110 | No |
| 118 | EHD2 |  |  | 4051 | -0.399 | -0.5143 | No |
| 119 | KLF5 |  |  | 4064 | -0.401 | -0.5150 | No |
| 120 | DNM1 |  |  | 4108 | -0.411 | -0.5219 | No |
| 121 | TSHZ2 |  |  | 4122 | -0.415 | -0.5227 | No |
| 122 | ATP2B1 |  |  | 4126 | -0.416 | -0.5216 | No |
| 123 | TRIM22 |  |  | 4138 | -0.419 | -0.5221 | No |
| 124 | TMEM98 |  |  | 4140 | -0.420 | -0.5205 | No |
| 125 | SPATS2L |  |  | 4148 | -0.422 | -0.5201 | No |
| 126 | PDLIM2 |  |  | 4180 | -0.432 | -0.5245 | No |
| 127 | HIC1 |  |  | 4189 | -0.434 | -0.5243 | No |
| 128 | MIR222HG |  |  | 4196 | -0.436 | -0.5237 | No |
| 129 | PLAC9 |  |  | 4197 | -0.437 | -0.5219 | No |
| 130 | AFDN |  |  | 4198 | -0.438 | -0.5201 | No |
| 131 | PDE5A |  |  | 4271 | -0.465 | -0.5324 | No |
| 132 | HTRA3 |  |  | 4274 | -0.466 | -0.5309 | No |
| 133 | ASPN |  |  | 4291 | -0.471 | -0.5321 | No |
| 134 | ABCA5 |  |  | 4293 | -0.471 | -0.5304 | No |
| 135 | PMAIP1 |  |  | 4312 | -0.476 | -0.5320 | No |
| 136 | EDNRA |  |  | 4361 | -0.489 | -0.5395 | No |
| 137 | TMEM167A |  |  | 4377 | -0.494 | -0.5404 | No |
| 138 | OMD |  |  | 4412 | -0.505 | -0.5451 | No |
| 139 | IFT20 |  |  | 4440 | -0.516 | -0.5483 | No |
| 140 | FHL3 |  |  | 4450 | -0.520 | -0.5479 | No |
| 141 | ARL1 |  |  | 4471 | -0.525 | -0.5497 | No |
| 142 | VCAN |  |  | 4491 | -0.537 | -0.5512 | No |
| 143 | SERTAD4 |  |  | 4508 | -0.545 | -0.5522 | No |
| 144 | ODF2L |  |  | 4524 | -0.553 | -0.5528 | No |
| 145 | ITGA5 |  |  | 4580 | -0.576 | -0.5614 | No |
| 146 | SCPEP1 |  |  | 4589 | -0.579 | -0.5606 | No |
| 147 | IGFBP7 |  |  | 4622 | -0.604 | -0.5644 | Yes |
| 148 | LOXL2 |  |  | 4624 | -0.605 | -0.5621 | Yes |
| 149 | S1PR3 |  |  | 4625 | -0.605 | -0.5596 | Yes |
| 150 | SNED1 |  |  | 4639 | -0.614 | -0.5596 | Yes |
| 151 | RBFOX2 |  |  | 4643 | -0.615 | -0.5577 | Yes |
| 152 | MAP1A |  |  | 4664 | -0.625 | -0.5590 | Yes |
| 153 | CDC42EP3 |  |  | 4669 | -0.627 | -0.5572 | Yes |
| 154 | NR3C1 |  |  | 4673 | -0.630 | -0.5552 | Yes |
| 155 | FZD2 |  |  | 4719 | -0.658 | -0.5614 | Yes |
| 156 | PLAGL1 |  |  | 4723 | -0.661 | -0.5593 | Yes |
| 157 | PKDCC |  |  | 4727 | -0.662 | -0.5571 | Yes |
| 158 | PYCR1 |  |  | 4747 | -0.676 | -0.5581 | Yes |
| 159 | ADAMTS12 |  |  | 4750 | -0.677 | -0.5557 | Yes |
| 160 | ACTN1 |  |  | 4775 | -0.695 | -0.5576 | Yes |
| 161 | PIM1 |  |  | 4783 | -0.699 | -0.5561 | Yes |
| 162 | IGFBP6 |  |  | 4800 | -0.715 | -0.5563 | Yes |
| 163 | PPIC |  |  | 4802 | -0.716 | -0.5535 | Yes |
| 164 | PCDH18 |  |  | 4803 | -0.717 | -0.5505 | Yes |
| 165 | BNC2 |  |  | 4819 | -0.730 | -0.5505 | Yes |
| 166 | EBF1 |  |  | 4831 | -0.738 | -0.5496 | Yes |
| 167 | PALLD |  |  | 4839 | -0.749 | -0.5479 | Yes |
| 168 | CNN2 |  |  | 4840 | -0.749 | -0.5448 | Yes |
| 169 | MDFIC |  |  | 4852 | -0.756 | -0.5438 | Yes |
| 170 | PDLIM7 |  |  | 4867 | -0.773 | -0.5434 | Yes |
| 171 | LIF |  |  | 4882 | -0.789 | -0.5429 | Yes |
| 172 | YIPF5 |  |  | 4908 | -0.820 | -0.5444 | Yes |
| 173 | P3H1 |  |  | 4912 | -0.825 | -0.5416 | Yes |
| 174 | NR2F2 |  |  | 4917 | -0.830 | -0.5390 | Yes |
| 175 | PLIN3 |  |  | 4921 | -0.833 | -0.5361 | Yes |
| 176 | NOTCH3 |  |  | 4936 | -0.852 | -0.5353 | Yes |
| 177 | SLC2A3 |  |  | 4942 | -0.859 | -0.5328 | Yes |
| 178 | TMEM263 |  |  | 4946 | -0.861 | -0.5298 | Yes |
| 179 | SEC24D |  |  | 4953 | -0.871 | -0.5274 | Yes |
| 180 | MIR22HG |  |  | 4968 | -0.885 | -0.5265 | Yes |
| 181 | KDELR3 |  |  | 4974 | -0.893 | -0.5238 | Yes |
| 182 | TMEM100 |  |  | 4979 | -0.897 | -0.5208 | Yes |
| 183 | COL8A1 |  |  | 5000 | -0.931 | -0.5209 | Yes |
| 184 | CLMP |  |  | 5008 | -0.948 | -0.5184 | Yes |
| 185 | CYTOR |  |  | 5022 | -0.978 | -0.5169 | Yes |
| 186 | EMP1 |  |  | 5023 | -0.979 | -0.5129 | Yes |
| 187 | PRKG1 |  |  | 5028 | -0.988 | -0.5096 | Yes |
| 188 | FLRT2 |  |  | 5029 | -0.989 | -0.5054 | Yes |
| 189 | NTN4 |  |  | 5032 | -0.991 | -0.5017 | Yes |
| 190 | PAWR |  |  | 5033 | -1.000 | -0.4976 | Yes |
| 191 | DLC1 |  |  | 5036 | -1.013 | -0.4938 | Yes |
| 192 | TENM2 |  |  | 5047 | -1.028 | -0.4915 | Yes |
| 193 | TWIST1 |  |  | 5060 | -1.052 | -0.4895 | Yes |
| 194 | COPZ2 |  |  | 5069 | -1.073 | -0.4866 | Yes |
| 195 | CTGF |  |  | 5070 | -1.073 | -0.4822 | Yes |
| 196 | RASL12 |  |  | 5074 | -1.084 | -0.4783 | Yes |
| 197 | CPED1 |  |  | 5078 | -1.090 | -0.4744 | Yes |
| 198 | GLT8D2 |  |  | 5079 | -1.094 | -0.4698 | Yes |
| 199 | GBP3 |  |  | 5080 | -1.094 | -0.4653 | Yes |
| 200 | EVA1A |  |  | 5081 | -1.098 | -0.4607 | Yes |
| 201 | ATP13A2 |  |  | 5089 | -1.120 | -0.4575 | Yes |
| 202 | LOX |  |  | 5092 | -1.128 | -0.4532 | Yes |
| 203 | PLXDC2 |  |  | 5096 | -1.138 | -0.4490 | Yes |
| 204 | PROCR |  |  | 5097 | -1.139 | -0.4443 | Yes |
| 205 | GLI1 |  |  | 5101 | -1.156 | -0.4401 | Yes |
| 206 | SRPX2 |  |  | 5108 | -1.176 | -0.4364 | Yes |
| 207 | COL11A1 |  |  | 5110 | -1.178 | -0.4317 | Yes |
| 208 | PERP |  |  | 5111 | -1.186 | -0.4268 | Yes |
| 209 | COL5A2 |  |  | 5112 | -1.189 | -0.4219 | Yes |
| 210 | LSP1 |  |  | 5113 | -1.190 | -0.4169 | Yes |
| 211 | CAVIN1 |  |  | 5116 | -1.195 | -0.4124 | Yes |
| 212 | NUPR1 |  |  | 5123 | -1.217 | -0.4085 | Yes |
| 213 | FAM114A1 |  |  | 5127 | -1.230 | -0.4040 | Yes |
| 214 | SYT1 |  |  | 5131 | -1.248 | -0.3994 | Yes |
| 215 | DUSP1 |  |  | 5138 | -1.269 | -0.3953 | Yes |
| 216 | ARL4C |  |  | 5143 | -1.279 | -0.3908 | Yes |
| 217 | SERPINF1 |  |  | 5158 | -1.351 | -0.3880 | Yes |
| 218 | GPX8 |  |  | 5171 | -1.418 | -0.3845 | Yes |
| 219 | BICC1 |  |  | 5172 | -1.423 | -0.3786 | Yes |
| 220 | CD248 |  |  | 5178 | -1.448 | -0.3736 | Yes |
| 221 | SOCS1 |  |  | 5184 | -1.475 | -0.3684 | Yes |
| 222 | COL12A1 |  |  | 5186 | -1.484 | -0.3625 | Yes |
| 223 | THBS1 |  |  | 5197 | -1.538 | -0.3581 | Yes |
| 224 | COL4A2 |  |  | 5198 | -1.545 | -0.3517 | Yes |
| 225 | TFPI |  |  | 5202 | -1.557 | -0.3458 | Yes |
| 226 | RARRES3 |  |  | 5206 | -1.582 | -0.3398 | Yes |
| 227 | MYOF |  |  | 5207 | -1.590 | -0.3332 | Yes |
| 228 | MFAP2 |  |  | 5208 | -1.592 | -0.3266 | Yes |
| 229 | NRP1 |  |  | 5211 | -1.609 | -0.3203 | Yes |
| 230 | BGN |  |  | 5216 | -1.654 | -0.3143 | Yes |
| 231 | OLFML3 |  |  | 5224 | -1.700 | -0.3086 | Yes |
| 232 | IGFBP4 |  |  | 5226 | -1.725 | -0.3016 | Yes |
| 233 | EGFR |  |  | 5230 | -1.765 | -0.2949 | Yes |
| 234 | SCN9A |  |  | 5232 | -1.797 | -0.2876 | Yes |
| 235 | DCN |  |  | 5233 | -1.798 | -0.2802 | Yes |
| 236 | LRP1 |  |  | 5234 | -1.801 | -0.2727 | Yes |
| 237 | SNAI2 |  |  | 5241 | -1.903 | -0.2660 | Yes |
| 238 | MXRA8 |  |  | 5244 | -1.939 | -0.2583 | Yes |
| 239 | CDH11 |  |  | 5245 | -1.943 | -0.2503 | Yes |
| 240 | CREB3L1 |  |  | 5246 | -1.960 | -0.2421 | Yes |
| 241 | PCOLCE |  |  | 5248 | -1.999 | -0.2340 | Yes |
| 242 | PDGFRB |  |  | 5253 | -2.045 | -0.2263 | Yes |
| 243 | MSRB3 |  |  | 5254 | -2.046 | -0.2178 | Yes |
| 244 | COLEC12 |  |  | 5256 | -2.134 | -0.2092 | Yes |
| 245 | CFH |  |  | 5257 | -2.141 | -0.2003 | Yes |
| 246 | CARMN |  |  | 5258 | -2.159 | -0.1913 | Yes |
| 247 | TRAC |  |  | 5259 | -2.169 | -0.1823 | Yes |
| 248 | DAB2 |  |  | 5261 | -2.217 | -0.1733 | Yes |
| 249 | FBN1 |  |  | 5263 | -2.234 | -0.1642 | Yes |
| 250 | COL4A1 |  |  | 5266 | -2.279 | -0.1552 | Yes |
| 251 | BHLHE41 |  |  | 5267 | -2.290 | -0.1457 | Yes |
| 252 | AHNAK |  |  | 5268 | -2.309 | -0.1361 | Yes |
| 253 | COL5A1 |  |  | 5274 | -2.455 | -0.1269 | Yes |
| 254 | LUM |  |  | 5277 | -2.507 | -0.1169 | Yes |
| 255 | ARHGAP29 |  |  | 5278 | -2.576 | -0.1062 | Yes |
| 256 | INHBA |  |  | 5282 | -2.697 | -0.0956 | Yes |
| 257 | SSPN |  |  | 5284 | -2.752 | -0.0844 | Yes |
| 258 | FN1 |  |  | 5287 | -2.866 | -0.0729 | Yes |
| 259 | PTCH1 |  |  | 5288 | -2.878 | -0.0609 | Yes |
| 260 | FOXS1 |  |  | 5291 | -3.324 | -0.0475 | Yes |
| 261 | COL3A1 |  |  | 5292 | -3.630 | -0.0325 | Yes |
| 262 | COL6A3 |  |  | 5293 | -3.722 | -0.0170 | Yes |
| 263 | COL1A1 |  |  | 5294 | -4.144 | 0.0002 | Yes |
Table: GSEA details [plain text format]

  

Fig 2: DOROSZKO\_U3054MG\_CLUSTER\_2: Random ES distribution      
 Gene set null distribution of ES for **DOROSZKO\_U3054MG\_CLUSTER\_2**
